# Caroline A. Evans, Phillip C. Wright, Josselin Noirel (Eds.): Mass spectrometry of proteins: methods and protocols

**DOI:** 10.1007/s00216-020-02580-1

**Published:** 2020-03-20

**Authors:** David Arnott

**Affiliations:** Genentech, Inc., 1 DNA Way, MS63, South San Francisco, CA 94080 USA


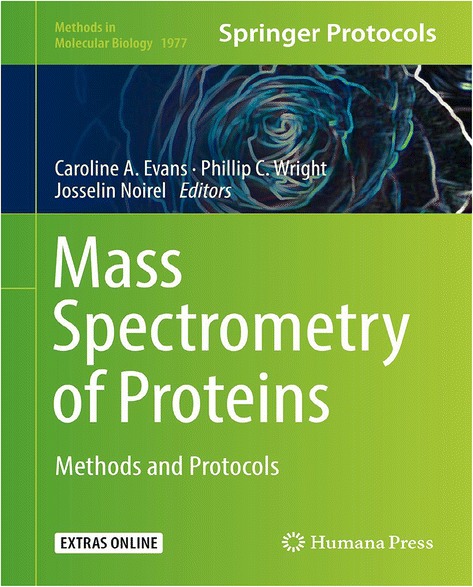


**Bibliography**

Mass spectrometry of proteins: methods and protocols

Series: Methods in molecular biology

Caroline A. Evans, Phillip C. Wright, and Josselin Noirel (Eds.)

Humana Press

ISBN: 978-1-4939-9231-7

Hardcover, 264 pages,

2019, €171.19

Book’s topic One reason for the success of proteomics is the versatility of mass spectrometers, their fruitful combination with all sorts of sample preparation workflows, and the wealth of information that is extractable from the resulting data. An ever-expanding list of analyses has entered the literature based on variations on protein extraction, enrichment, and manipulation. Increasingly complex data sets bring attendant complexity in their interpretation. Even experienced mass spectrometrists can find themselves tasked with unfamiliar analyses, success often depending on critical but not obvious details. *Mass spectrometry of proteins* contains protocol-focused chapters written by experts in their use and includes exactly these sorts of critical details. Recognizing the importance of informatics, it also treats aspects of experimental design and data analysis in similar detail.

Contents The book contains two parts: “New Biological Insights from Technological Breakthroughs” and “Dealing with Proteomics Data in a Big Data Era”. Part I covers experimental applications including those enabled by mass spectrometry hardware like “SWATH” (Chapter 1), or “PRM” (ch 3), or innovative sample preparation, such as chemical derivatization (ch 2), or biotin-exchange (ch 6). Some, like chapter 4 on phosphopeptide enrichment, and chapter 9 on protein complexes cover well-known workflows, but are distinguished by their detailed protocols and key considerations in their use. Part II is informatics-oriented and spans topics from the general, like experimental design (ch 11) or archiving and dissemination of quantitative data (ch 14), to specific analyses like isobaric mass tags (ch 13) and programming platforms and pipelines (chs 15 and 16).

Comparison with the existing literature Biological mass spectrometry covers enough ground that editors must find ways to limit their scope. Books like *Protein analysis using mass spectrometry: accelerating biotherapeutics from lab to patient* (Lee and Ji Eds.), *Analysis of protein post-translational modifications by mass spectrometry* (Griffiths and Unwin Eds.), and *Sample preparation in mass spectrometry* (Ivanov and Lazarev Eds.) cover some of the same ground as *Mass spectrometry of proteins* and achieve greater depth on the subjects their titles indicate. *Mass spectrometry of proteins* is distinct, however, in its inclusion of methodology for the wet lab, instrument lab, and informatics, and excels in the specificity of its protocols. Textbooks such as Pradip K. Ghosh’s *Introduction to protein mass spectrometry* provide a more coherent overview of the field but cannot match the level of practical detail provided.

Critical assessment A quibble is that although titled *Mass spectrometry of proteins*, there is probably not a single mass spectrum of a protein described in the whole book. All of the analyses are “bottom-up” approaches where proteins are digested enzymatically, and the resulting oligopeptides analyzed by mass spectrometry. In years past, it might have gone without saying that proteomics was done this way, but there are now enough examples of “top down” and “native” protein mass spectrometry to make the distinction important. That aside, there is something here for most practitioners of biological mass spectrometry, and a few chapters are particularly strong. I would recommend chapter 6 on the dynamics of protein acetylation or chapter 9 on purifying protein complexes to anyone attempting such analyses. And chapter 7 on cysteine reduction and alkylation is a welcome compilation of knowledge concerning a ubiquitous but underappreciated part of proteomic workflows that is nonetheless critical for success. The chapters on data analysis are timely, given the evolution of proteomics, and the links to R scripts, Python applications, and sample data are useful to both those new to the field (for general considerations) and those with the expertise in programming or statistics who will be able to use and adapt the provided tools.

Readership recommendation and summary *Mass spectrometry of proteins* is written for the practicing scientist, and its strength in is the level of detail provided. Such books cannot hope to be comprehensive, so its usefulness will depend on the interests of the reader intersecting with specific chapters, but there are topics here of broad interest, such as the ones on cysteine reduction and S-alkylation, experimental design, and protein inference, along with more esoteric topics like metaproteomics and palmitoylation. And I would recommend Felderspiel and Cristea’s chapter on protein co-immunoprecipitation as required reading for even researchers experienced in this widely performed analysis. Readers on familiar ground with Part I are likely to be different from those comfortable with Part II, but it is a positive development to find both laboratory protocols and consideration for experimental design and data analysis brought together.

